# A Case Series of Spinal Infections Following COVID-19: A Delayed Complication

**DOI:** 10.7759/cureus.29272

**Published:** 2022-09-17

**Authors:** Fadzrul Abbas Mohamed Ramlee, Mohd Hezery Bin Harun, Vinodharan Nagaretnam, Teck Siang Lim, Hasry Faris Aris, Chor Ngee Tan

**Affiliations:** 1 Orthopaedic Surgery, Hospital Pengajar Universiti Putra Malaysia, Serdang, MYS; 2 Orthopaedics and Traumatology, Hospital Pengajar Universiti Putra Malaysia, Serdang, MYS; 3 Spine Surgery, Hospital Pengajar Universiti Putra Malaysia, Serdang, MYS; 4 Orthopaedics, Hospital Putrajaya, Putrajaya, MYS

**Keywords:** latent tb infection, fallout of covid-19, opportunistic infections of the spine, sars-cov-2, covid-19, covid- 19 infection, pyogenic spondylodiscitis, treatment of spinal epidural abscess, spine tuberculosis

## Abstract

Spinal infection in the form of tuberculous vertebral osteomyelitis or pyogenic spondylodiscitis is a commonly associated state of an immunodeficient host from various pathologies. For example, secondary infections can be seen following coronavirus disease 2019 (COVID-19). We report three cases of different forms of spinal infections that occurred as delayed complications to recent COVID-19 infection. The first case is a 60-year-old female who was diagnosed with an epidural abscess presenting with severe back pain and bilateral lower limb weakness. The second case is an elderly male who was diagnosed with L3/L4 spondylodiscitis and presented with predominantly back pain and minimal leg symptom. The final case is a young female who was diagnosed with severe T5 tuberculous spondylitis and presented with a complete sensory and motor deficit from T5 below. All patients showed good improvement after surgery and antibiotic therapy. Patients treated for COVID-19 are at risk of spinal infection development due to multiple pathophysiologies. Treatment of these various forms of spinal infection remains difficult, and we encourage physicians to be vigilant for the development of these complications post COVID-19 infection.

## Introduction

Coronavirus disease 2019 (COVID-19) infection brought about tremendous changes in the world. It remains a challenge to the medical community, for it has been a constant uphill battle managing the myriad complications that occur as a result of this deadly virus infection. COVID-19 causes damage to the endothelium of vessels in the lung, giving rise to its pulmonary complications [1,2]. Recently, the widespread systemic involvement of the virus is gaining notice. One such problem is the spine and the neurological system. Epidural and retropharyngeal abscesses have been reported in tandem with the COVID-19 infection. The epidural abscesses plague patients with paraplegia or quadriparesis [2-4]. We would like to share our experience of managing three patients who developed different types of spine-related infections shortly after recovering from COVID-19 infection.

## Case presentation

Case 1

**Figure 1:**
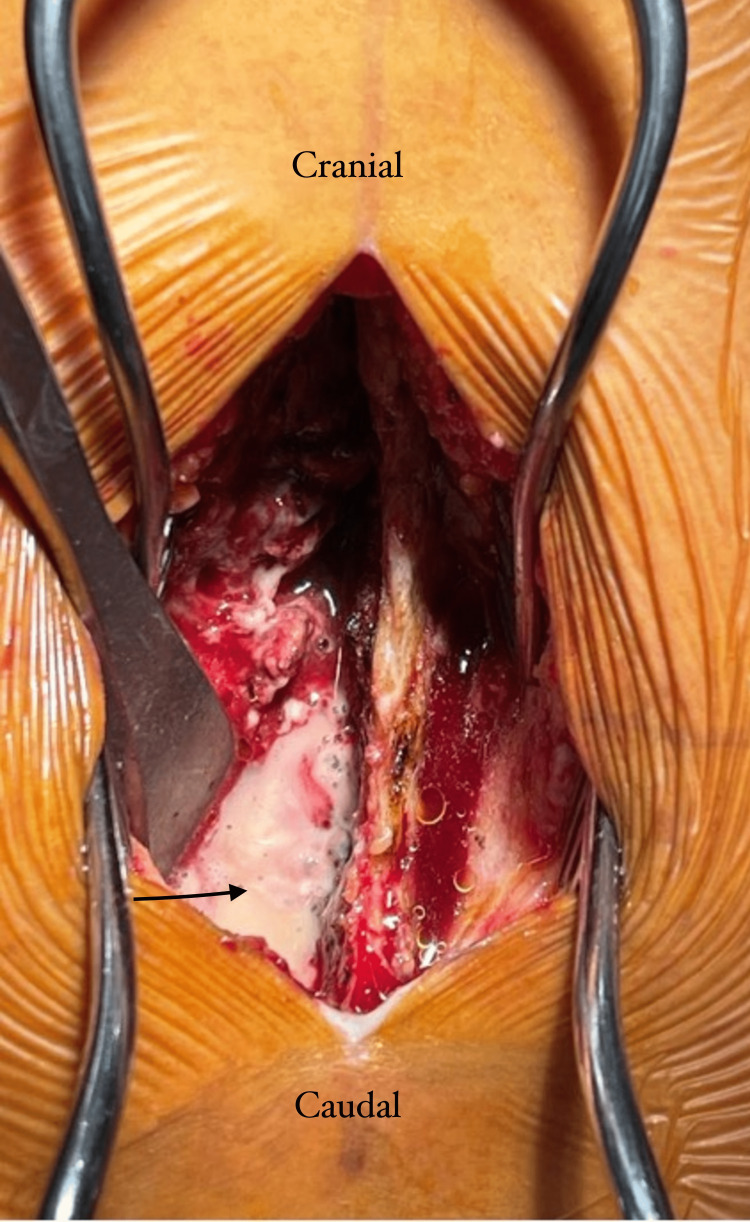
Intraoperative images showing an epidural abscess collection. The arrow marks the abscess collection at the left hemilaminectomy area.

A 60-year-old female, with a history of COVID-19 infection 10 weeks prior to presentation, complained of sudden onset lower back pain associated with bilateral lower extremity weakness, without urinary or bowel incontinence. She also denied prior trauma or previous spine infections. She complained of being lethargic and having loose stools for a week before seeking medical attention for her condition. She was afebrile and had an L4 midline spine tenderness at the L4 vertebra, while the L2-L4 motor strength was decreased; she had intact sensation and reflexes. Laboratory investigation revealed leukocytosis and raised C-reactive protein (CRP), while her blood culture and sensitivity grew methicillin-sensitive *Staphylococcus aureus* (MSSA). A whole-spine magnetic resonance imaging (MRI) with contrast revealed subcutaneous L1-L5 edema with an L3-S2 intramuscular collection within the bilateral erector spinae muscles. An L4/L5 epidural space collection was seen at the right L4/L5 facet joint and also compressing the cauda equina resulting in a narrowed spinal canal (Figure 1). She was started on empirical intravenous piperacillin and tazobactam and later changed to cefazolin, following which she underwent an L4/L5 decompressive laminectomy, and debridement. She then completed six weeks of intravenous cloxacillin based on the intraoperative specimen, which grew MSSA. During the postoperative period of two months, she was able to ambulate without support or back pain. 

Case 2

**Figure 2:**
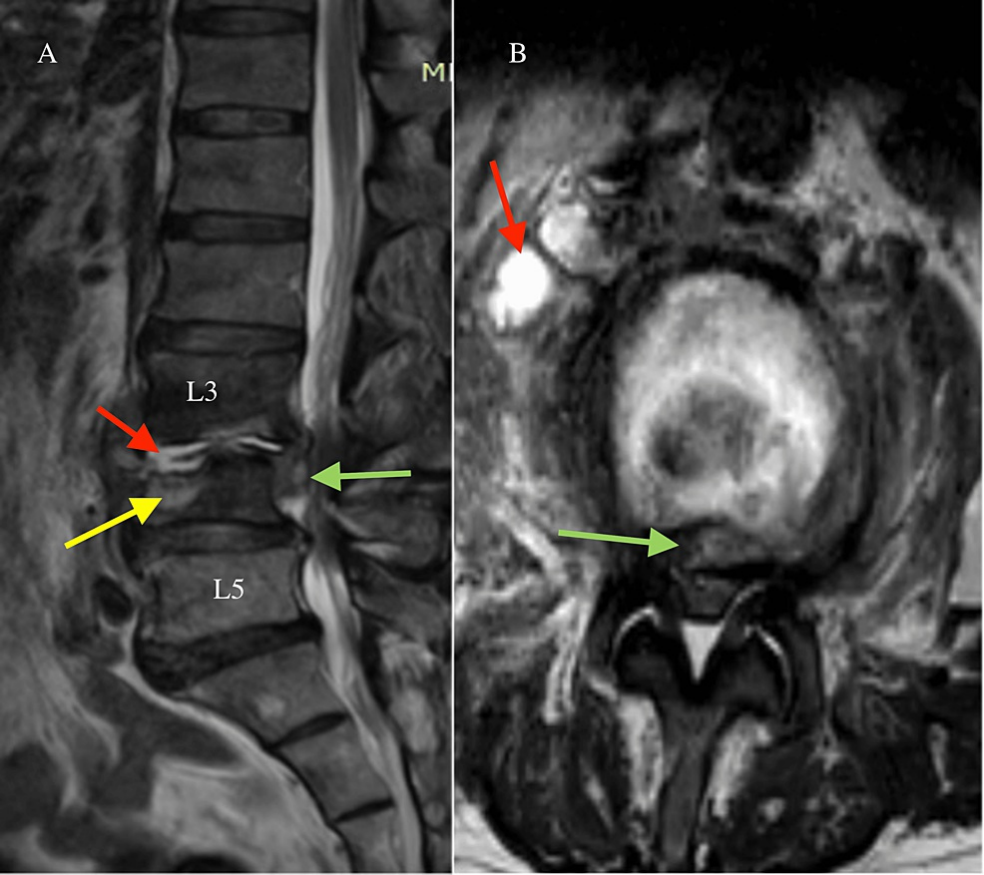
(A) Sagittal T2-weighted and (B) axial T2-weighted L3/L4 MRIs show an increased signal intensity of the L3/L4 intervertebral disc and surrounding tissues and demonstrate significant vertebral destruction and the abscess collection. The red arrows indicate the abscess collection at the disc level and at the anterolateral aspect of the disc. The green arrows point to the epidural abscess that was causing a severe stenosis of the spinal canal. The yellow arrow indicates the L5 bone destruction and edema.

The second patient was a 69-year-old male with underlying ischemic heart disease, prostate cancer with bone metastasis, and a history of COVID-19 infection three months prior complicated by deep vein thrombosis. He presented with new onset lower back pain for approximately two months. He denied any recent trauma or previous infections of the spine. He was afebrile and hemodynamically stable; however, there was instability-related spinal tenderness along the lower lumbar region. A neurological examination revealed an intact lower extremity motor power and sensation. He had mild leukocytosis and an elevated CRP, while the MRI revealed an abscess collection and high signal intensity of the intervertebral disc at the level of the L3/L4 and L4/L5 vertebrae (Figure 2). He underwent surgery for debridement of the affected spinal levels and stabilization via a transforaminal lumbar interbody fusion (TLIF). Intraoperatively, it was noted that there was unhealthy disc material at L3/L4 and L4/L5 as well as erosion of the superior endplate of the L4 vertebral body. Intraoperative specimens grew no organisms; however, histopathological examination revealed features of chronic inflammation while ruling out malignancy. He was then treated with intravenous ceftriaxone for a duration of six weeks. At a postoperative period of approximately two months, he had minimal back pain and was able to walk independently with a walker.

Case 3

**Figure 3:**
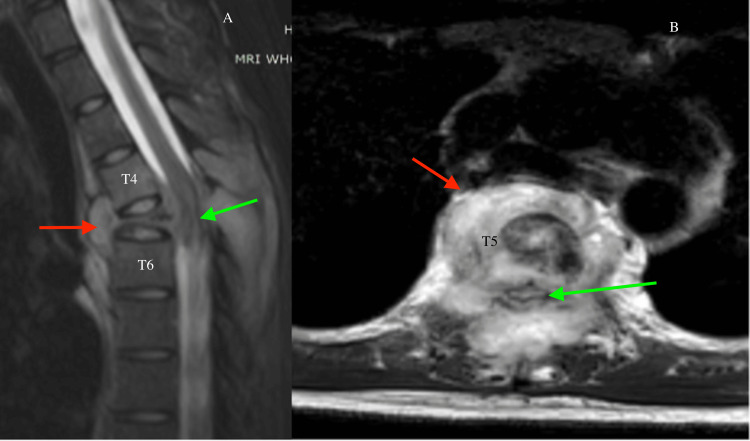
(A) Sagittal T2-weighted MRI and (B) axial T2-weighted MRI show severe destruction of the T5 vertebral body and posterior elements with a surrounding abscess collection. The red arrows point to the abscess collection below the anterior longitudinal ligament and over the T5 vertebra. The green arrows demonstrate the epidural abscess collection at the T5 vertebra with cord edema.

**Figure 4:**
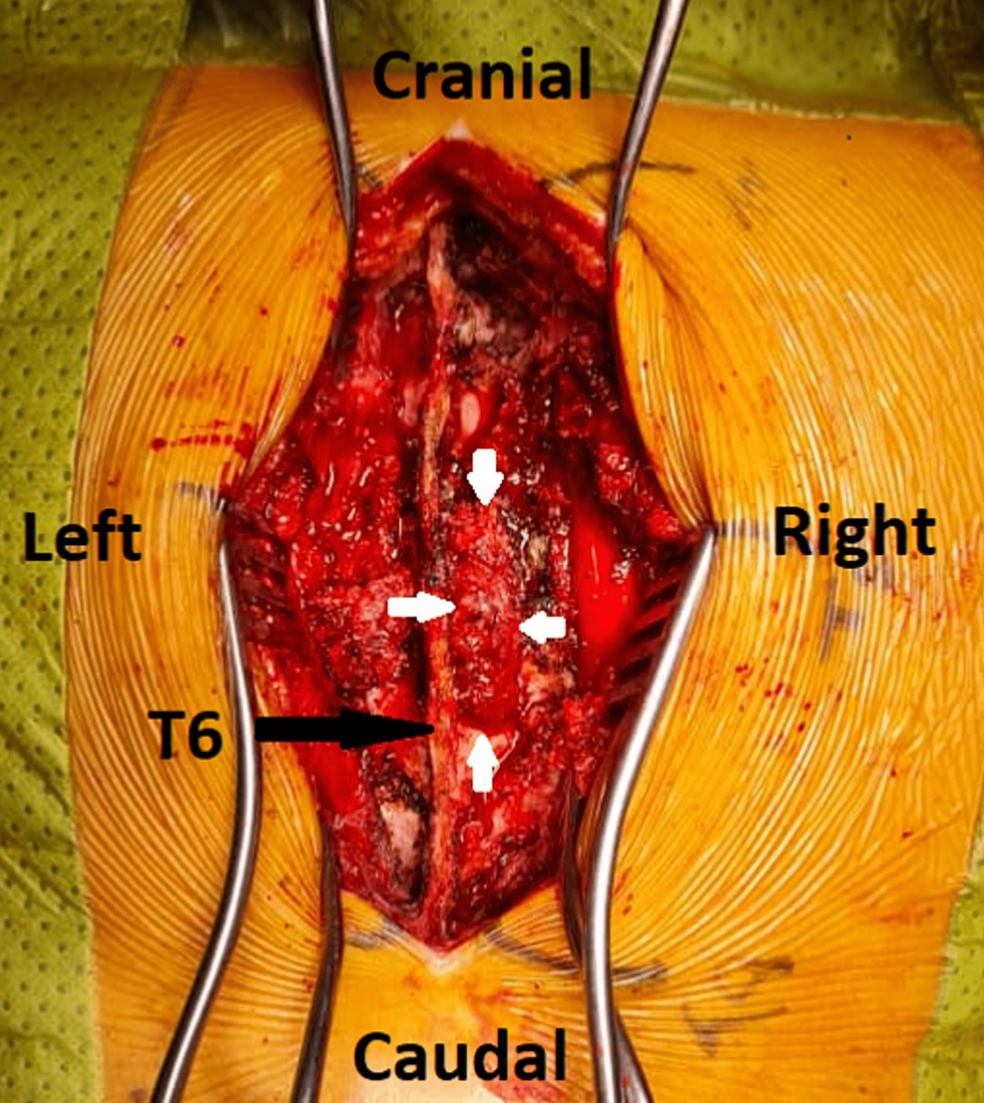
A brownish-grey, 9 cm x 4 cm multilobulated soft tissue granulomatous mass is shown protruding through an opening in the right T5 lamina which has undergone destruction by Mycobactrium tuberculosis (white arrows).

A 24-year-old female presented with back pain for the past three months and the sudden onset of bilateral lower extremity weakness, numbness, and urinary and bowel incontinence. She denied any traumatic events leading to her condition or any previous history of spine-related infections. She had no previous comorbidities but revealed a recent history of COVID-19 infection requiring only home quarantine about three months prior to her presentation. She had no known contact with tuberculosis (TB) patients in the past. On neurological examination, she had a complete absence of motor function and sensation below the T4 level. Her MRI revealed destruction of the T5 vertebral body and posterior elements with a subligamentous collection sparing the intervertebral discs, with resultant spinal canal stenosis and nerve root impingement (Figure 3). She underwent debridement surgery, T5 corpectomy and T2-T7 posterior instrumentation. Intraoperatively, there was severe destruction not only of the T5 vertebrae but also of the T4/T5 posterior elements (Figure 4). While cultures of intraoperative specimens grew no organisms, histopathological examination revealed chronic necrotizing granulomatous inflammation, highly suggestive of *Mycobacterium tuberculosis*. She was started on anti-TB treatment and enrolled in a rehabilitation program. At six months after surgery, she was able to independently ambulate with a walker and was continent.

## Discussion

It is interesting to note that a recent COVID-19 infection is the common factor seen in all three patients who developed spine-related infections. COVID-19 has been implicated in multi-system aberrations apart from its primary target of the lungs [4]. Opportunistic infections occurring secondarily after COVID-19 were reported in 27% of patients who were hospitalized in Wuhan, China [5]. Choudury et al. described exhaustion of the cellular-mediated response, which results in the reduction of the CD4 and CD8+ cells involved in initiating humoral and cytotoxic responses against viral pathogens [2]. This implies the increased susceptibility of patients with COVID-19 to be debilitated by opportunistic infections in the face of a blunted immune response. Immunomodulating drugs such as tocilizumab, which are prescribed for their off-label use to offset cytokine release storm, are in turn causing immunodeficient responses against superimposed bacterial infection [5]. Patients also present with superimposed bacterial infections even without the immunomodulating drug being described such as seen in our patients.

A spinal epidural abscess (SEA) is commonly seen in a patient who has or has had an adjacent spondylodiscitis. It also can occur after and adjacent to an infected spine surgery or via a hematogenous spread [6]. SEAs occur mainly in patients who are immunocompromised or are intravenous drug abusers. A SEA can also occur in patients who have indwelling catheters, chronic kidney disease, diabetes mellitus, obesity, and a history of alcohol abuse [1,2]. As such, primary SEA accounts for only 20% of all documented epidural abscesses in the literature [1,2]. The majority of SEAs occur in the lumbar spine, although in patients presenting after COVID-19, it has been reported in the cervical and thoracic regions, giving rise to quadriparesis and poor neurological outcomes [1,2]. 

The most common organism involved in opportunistic infections of the spine causing SEA is *Staphylococcus aureus*, MSSA as seen in our patient, followed closely by other *Staphylococcus* bacteria [1,2,4,5]. MRI is the diagnostic imaging of choice, which reveals a hyperintense lesion on T2-weighting encroaching into the epidural space as seen in our patients, which also encroaches and then displaces the cauda equina [1,2,4,5].

Besides SEA, spondylodiscitis is another possible common complication that can arise in the spinal structures following COVID-19 infection. Such an occurrence may pose additional morbidity and mortality, as it is a serious condition attributed to a dysbalanced immune response with immunosuppression and it can occur following COVID-19 infection. Naderi et al. reported a case of an elderly male who was diagnosed with COVID-19 pneumonia and eventually developed paraparesis and paraplegia due to a thoracic multilevel spondylodiscitis and epidural abscess. He succumbed to this condition due to multiorgan failure despite undergoing surgery to evacuate the abscess and decompress the spinal cord [7]. In another case reported by Erok et al., a middle-aged male who was diagnosed with COVID-19 and underwent quarantine was admitted for persistent right-sided leg pain. Further investigation revealed spondylodiscitis at the lumbar spine, as well as a right-sided psoas abscess. He underwent aspiration of the abscess, which revealed no growth. Eventually, however, worsening of spinal elements was seen in serial MRIs three weeks later [8]. This highlights the importance of always suspecting secondary spinal infections in COVID-19 patients with neuromuscular symptoms. 

Besides superimposed bacterial infections of the spine because of immunosuppression from COVID-19 infection, co-infection and re-activation of a chronic granulomatous organism such as TB should always be considered, especially if the clinical findings are not suggestive of common bacterial infections in such patients. As a result of immunosuppression from a prior COVID-19 infection, it is highly possible for a latent TB infection to be activated, leading to an active manifestation of the disease in this group of patients, as also seen in one of the reported cases above. Such similar scenarios have also been reported by Burda et. al. whereby a middle-aged man who recovered from COVID-19 infection presented with symptoms of an acute progressive encephalopathy three weeks later and was then diagnosed as TB based on a positive *Mycobacterium* polymerase chain reaction (PCR) test despite all other tests repeatedly yielding no growth [9]. This reinforces the importance of actively ruling out a granulomatous infection in all suspected cases of spine infections following COVID-19 as the findings may be easily masked. Detection of an active TB infection in COVID-19 patients is important as the outcome may be poor, especially if TB treatment is delayed [10].

Having established the causative factors of all spinal-related infections following or during COVID-19 infection, the mainstay of treatment remains appropriate antibiotics or specific anti-TB drugs as well as urgent surgical decompression of the spinal cord and/or neural elements. Urgent decompression of the spine is crucial as the recovery of the neurological status correlates with the duration of neurological deficit prior to surgery [1,2].

## Conclusions

COVID-19 infection may cause delayed-onset complications to the host with risks of secondary bacterial infection or activation of a latent TB infection in extrapulmonary sites, of which spinal elements are commonly involved. Understanding the pathophysiology of COVID-19 infection is crucial to enable treating clinicians to embark on the correct methods to identify the accurate causative factors in patients presenting with neuromuscular-related illnesses following COVID-19 infection. This may also help clinicians to actively screen for such possible complications in all patients who have contracted the virus. This may enable them to halt the progression of the disease at the initial stage, which can lead to reduced morbidity and mortality. 
